# Second Competition on Software Testing: Test-Comp 2020

**DOI:** 10.1007/978-3-030-45234-6_25

**Published:** 2020-03-13

**Authors:** Dirk Beyer

**Affiliations:** 8 University of Paderborn, Paderborn, Germany; 9 ICREA, Open University of Catalonia, Barcelona, Spain; LMU Munich, Munich, Germany

**Keywords:** Software Testing, Test-Case Generation, Competition, Software Analysis, Software Validation, Test Validation, Test-Comp, Benchmarking, Test Coverage, Bug Finding, BenchExec, TestCov

## Abstract

This report describes the 2020 Competition on Software Testing (Test-Comp), the 2$^{\text {nd}}$ edition of a series of comparative evaluations of fully automatic software test-case generators for C programs. The competition provides a snapshot of the current state of the art in the area, and has a strong focus on replicability of its results. The competition was based on 3 230 test tasks for C programs. Each test task consisted of a program and a test specification (error coverage, branch coverage). Test-Comp 2020 had 10 participating test-generation systems.

## 1 Introduction

Software testing is as old as software development itself, because the most straightforward way to find out if the software works is to execute it. In the last few decades the tremendous breakthrough of fuzzers [1], theorem provers [40], and satisfiability-modulo-theory (SMT) solvers [21] have led to the development of efficient tools for automatic test-case generation. For example, symbolic execution and the idea to use it for test-case generation [33] exists for more than 40 years, yet, efficient implementations (e.g., Klee [16]) had to wait for the availability of mature constraint solvers. Also, with the advent of automatic software model checking, the opportunity to extract test cases from counterexamples arose (see Blast [9] and JPF [41]). In the following years, many techniques from the areas of model checking and program analysis were adapted for the purpose of test-case generation and several strong hybrid combinations have been developed [24].

There are several powerful software test generators available [24], but they were difficult to compare. For example, a recent study [11] first had to develop a framework that supports to run test-generation tools on the same program source code and to deliver test cases in a common format for validation. Furthermore, there was no widely distributed benchmark suite available and neither input programs nor output test suites followed a standard format. In software verification, the competition SV-COMP [3] helped to overcome the problem: the competition community developed standards for defining nondeterministic functions and a

[1] http://lcamtuf.coredump.cx/afl/

language to write specifications (so far for C and Java programs) and established a standard exchange format for the output (witnesses). A competition event with high visibility can foster the transfer of theoretical and conceptual advancements in the area of software testing into practical tools.

The annual Competition on Software Testing (Test-Comp) [4,5] [2] is the showcase of the state of the art in the area, in particular, of the effectiveness and efficiency that is currently achieved by tool implementations of the most recent ideas, concepts, and algorithms for fully automatic test-case generation. Test-Comp uses the benchmarking framework BenchExec [12], which is already successfully used in other competitions, most prominently, all competitions that run on the StarExec infrastructure [39]. Similar to SV-COMP, the test generators in Test-Comp are applied to programs in a fully automatic way. The results are collected via BenchExec's XML results format, and transformed into tables and plots in several formats.[3] All results are available in artifacts at Zenodo (Table 3).

**Competition Goals.** In summary, the goals of Test-Comp are the following:

- Establish *standards* for software test generation. This means, most prominently, to develop a standard for marking input values in programs, define an exchange format for test suites, and agree on a specification language for test-coverage criteria, and define how to validate the resulting test suites.
- Establish a set of *benchmarks* for software testing in the community. This means to create and maintain a set of programs together with coverage criteria, and to make those publicly available for researchers to be used in performance comparisons when evaluating a new technique.
- Provide an overview of *available tools* for test-case generation and a snapshot of the state-of-the-art in software testing to the community. This means to compare, independently from particular paper projects and specific techniques, different test-generation tools in terms of effectiveness and performance.
- Increase the visibility and credits that *tool developers* receive. This means to provide a forum for presentation of tools and discussion of the latest technologies, and to give the students the opportunity to publish about the development work that they have done.
- Educate PhD students and other participants on how to set up performance experiments, packaging tools in a way that supports replication, and how to perform *robust and accurate research experiments*.
- Provide *resources* to development teams that do not have sufficient computing resources and give them the opportunity to obtain results from experiments on large benchmark sets.

**Related Competitions.** In other areas, there are several established competitions. For example, there are three competitions in the area of software verification: (i) a competition on automatic verifiers under controlled resources (SV-COMP [3]), (ii) a competition on verifiers with arbitrary environments (RERS [27]), and (iii) a competition on interactive verification (VerifyThis [28]). An overview of

[2] https://test-comp.sosy-lab.org

[3] https://test-comp.sosy-lab.org/2020/results/

16 competitions in the area of formal methods was presented at the TOOLympics events at the conference TACAS in 2019 [1]. In software testing, there are several competition-like events, for example, the DARPA Cyber Grand Challenge [38] [4], the IEEE International Contest on Software Testing [5], the Software Testing World Cup [6], and the Israel Software Testing World Cup [7]. Those contests are organized as on-site events, where teams of people interact with certain testing platforms in order to achieve a certain coverage of the software under test. There are two competitions for automatic and off-site testing: Rode0day [8] is a competition that is meant as a continuously running evaluation on bug-finding in binaries (currently Grep and SQLite). The unit-testing tool competition [32] [9] is part of the SBST workshop and compares tools for unit-test generation on Java programs. There was no comparative evaluation of automatic test-generation tools for whole C programs in source-code, in a controlled environment, and Test-Comp was founded to close this gap [4]. The results of the first edition of Test-Comp were presented as part of the TOOLympics 2019 event [1] and in the Test-Comp 2019 competition report [5].

## 2 Definitions, Formats, and Rules

Organizational aspects such as the classification (automatic, off-site, reproducible, jury, traning) and the competition schedule is given in the initial competition definition [4]. In the following we repeat some important definitions that are necessary to understand the results.

**Test Task.** A *test task* is a pair of an input program (program under test) and a test specification. A *test run* is a non-interactive execution of a test generator on a single test task, in order to generate a test suite according to the test specification. A *test suite* is a sequence of test cases, given as a directory of files according to the format for exchangeable test-suites.[10]

**Execution of a Test Generator.** Figure 1 illustrates the process of executing one test generator on the benchmark suite. One test run for a test generator gets as input (i) a program from the benchmark suite and (ii) a test specification (find bug, or coverage criterion), and returns as output a test suite (i.e., a set of test cases). The test generator is contributed by a competition participant. The test runs are executed centrally by the competition organizer. The test validator takes as input the test suite from the test generator and validates it by executing the program on all test cases: for bug finding it checks if the bug is exposed and for coverage it reports the coverage. We use the tool TestCov [14] [11] as test-suite validator.

[4] https://www.darpa.mil/program/cyber-grand-challenge/
[5] http://paris.utdallas.edu/qrs18/contest.html
[6] http://www.softwaretestingworldcup.com/
[7] https://www.inflectra.com/Company/Article/480.aspx
[8] https://rode0day.mit.edu/
[9] https://sbst19.github.io/tools/
[10] https://gitlab.com/sosy-lab/software/test-format/
[11] https://gitlab.com/sosy-lab/software/test-suite-validator

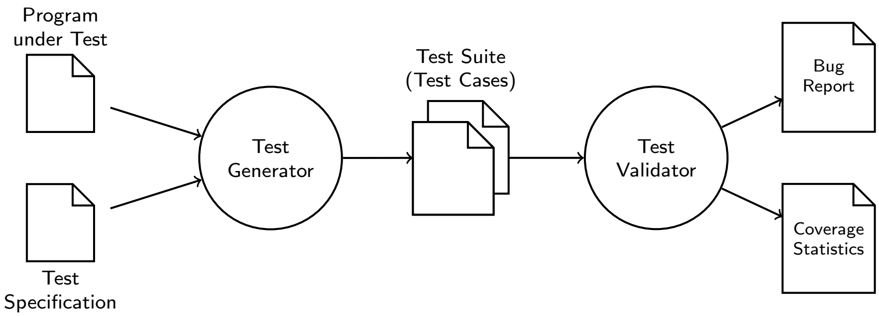

Fig. 1: Flow of the Test-Comp execution for one test generator

Table 1: Coverage specifications used in Test-Comp 2020 (same as in 2019)

| Formula | Interpretation |
|---|---|
| `COVER EDGES(@CALL(__VERIFIER_error))` | The test suite contains at least one test that executes function `__VERIFIER_error`. |
| `COVER EDGES(@DECISIONEDGE)` | The test suite contains tests such that all branches of the program are executed. |

**Test Specification.** The specification for testing a program is given to the test generator as input file (either `properties/coverage-error-call.prp` or `properties/coverage-branches.prp` for Test-Comp 2020).

The definition `init(main())` is used to define the initial states of the program under test by a call of function `main` (with no parameters). The definition `FQL(f)` specifies that coverage definition `f` should be achieved. The FQL (FShell query language [26]) coverage definition `COVER EDGES(@DECISIONEDGE)` means that all branches should be covered, `COVER EDGES(@BASICBLOCKENTRY)` means that all statements should be covered, and `COVER EDGES(@CALL(__VERIFIER_error))` means that calls to function `__VERIFIER_error` should be covered. A complete specification looks like: `COVER( init(main()), FQL(COVER EDGES(@DECISIONEDGE)) )`.

Table 1 lists the two FQL formulas that are used in test specifications of Test-Comp 2020; there was no change from 2019. The first describes a formula that is typically used for bug finding: the test generator should find a test case that executes a certain error function. The second describes a formula that is used to obtain a standard test suite for quality assurance: the test generator should find a test suite for branch coverage.

**License and Qualification.** The license of each participating test generator must allow its free use for replication of the competition experiments. Details on qualification criteria can be found in the competition report of Test-Comp 2019 [5].

## 3 Categories and Scoring Schema

**Benchmark Programs.** The input programs were taken from the largest and most diverse open-source repository of software verification tasks [12], which is also used by SV-COMP [3]. As in 2019, we selected all programs for which the following properties were satisfied (see issue on GitHub [13] and report [5]):

1. compiles with `gcc`, if a harness for the special methods [14] is provided,
2. should contain at least one call to a nondeterministic function,
3. does not rely on nondeterministic pointers,
4. does not have expected result 'false' for property 'termination', and
5. has expected result 'false' for property 'unreach-call' (only for category *Error Coverage*).

This selection yielded a total of 3 230 test tasks, namely 699 test tasks for category *Error Coverage* and 2 531 test tasks for category *Code Coverage*. The test tasks are partitioned into categories, which are listed in Tables 6 and 7 and described in detail on the competition web site.[15] Figure 2 illustrates the category composition.

**Category Error-Coverage.** The first category is to show the abilities to discover bugs. The programs in the benchmark set contain programs that contain a bug. Every run will be started by a batch script, which produces for every tool and every test task (a C program together with the test specification) one of the following scores: 1 point, if the validator succeeds in executing the program under test on a generated test case that explores the bug (i.e., the specified function was called), and 0 points, otherwise.

**Category Branch-Coverage.** The second category is to cover as many branches of the program as possible. The coverage criterion was chosen because many test-generation tools support this standard criterion by default. Other coverage criteria can be reduced to branch coverage by transformation [25]. Every run will be started by a batch script, which produces for every tool and every test task (a C program together with the test specification) the coverage of branches of the program (as reported by TestCov [14]; a value between 0 and 1) that are executed for the generated test cases. The score is the returned coverage.

**Ranking.** The ranking was decided based on the sum of points (normalized for meta categories). In case of a tie, the ranking was decided based on the run time, which is the total CPU time over all test tasks. Opt-out from categories was possible and scores for categories were normalized based on the number of tasks per category (see competition report of SV-COMP 2013 [2], page 597).

[12] https://github.com/sosy-lab/sv-benchmarks
[13] https://github.com/sosy-lab/sv-benchmarks/pull/774
[14] https://test-comp.sosy-lab.org/2020/rules.php
[15] https://test-comp.sosy-lab.org/2020/benchmarks.php

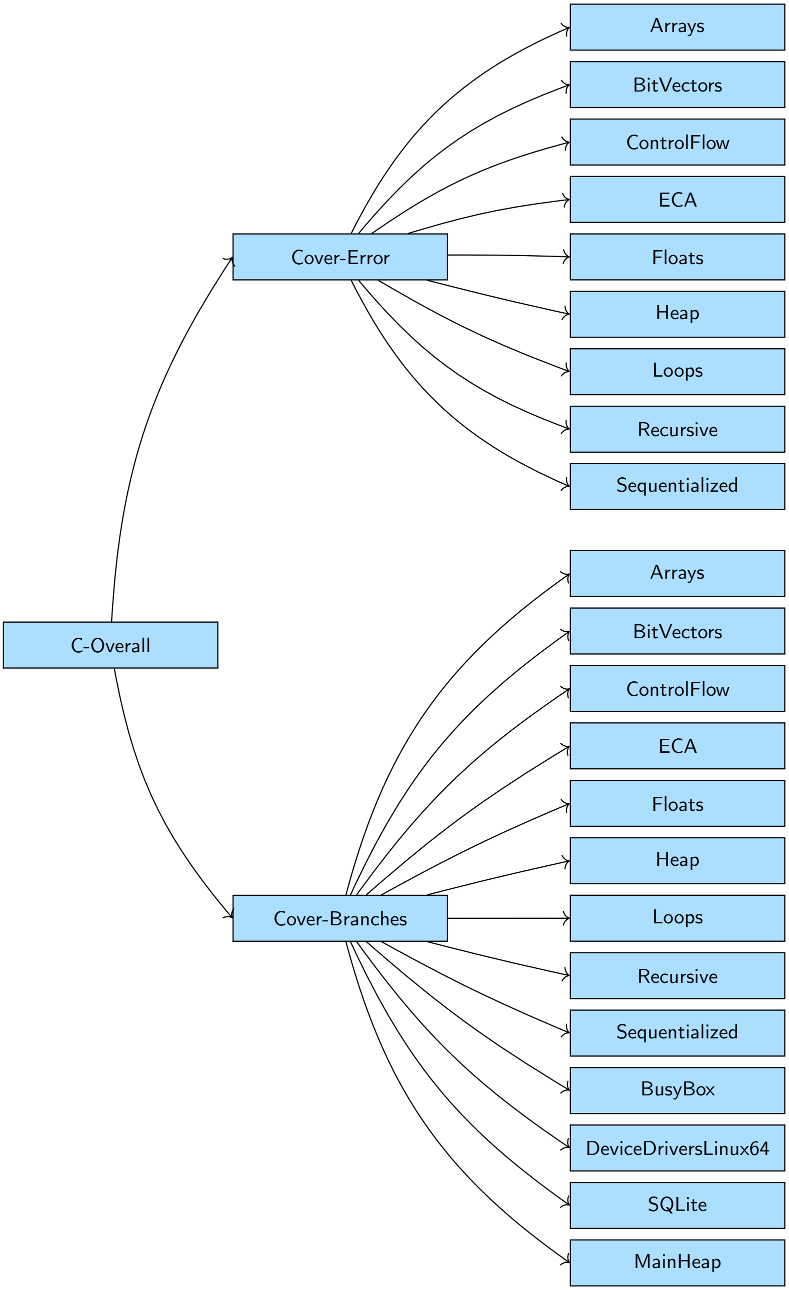

Fig. 2: Category structure for Test-Comp 2020

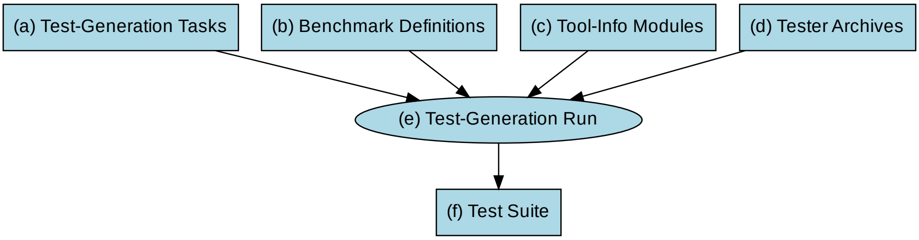

Fig. 3: Test-Comp components and the execution flow

Table 2: Publicly available components for replicating Test-Comp 2020

| Component | Fig. 3 | Repository | Version |
|---|---|---|---|
| Test-Generation Tasks | (a) | `github.com/sosy-lab/sv-benchmarks` | `testcomp20` |
| Benchmark Definitions | (b) | `gitlab.com/sosy-lab/test-comp/bench-defs` | `testcomp20` |
| Tool-Info Modules | (c) | `github.com/sosy-lab/benchexec` | `2.5.1` |
| Tester Archives | (d) | `gitlab.com/sosy-lab/test-comp/archives-2020` | `testcomp20` |
| Benchmarking | (e) | `github.com/sosy-lab/benchexec` | `2.5.1` |
| Test-Suite Format | (f) | `gitlab.com/sosy-lab/software/test-format` | `testcomp20` |

## 4 Reproducibility

In order to support independent replication of the Test-Comp experiments, we made all major components that are used for the competition available in public version repositories. An overview of the components that contribute to the reproducible setup of Test-Comp is provided in Fig. 3, and the details are given in Table 2. We refer to the report of Test-Comp 2019 [5] for a thorough description of all components of the Test-Comp organization and how we ensure that all parts are publicly available for maximal replicability.

In order to guarantee long-term availability and immutability of the test-generation tasks, the produced competition results, and the produced test suites, we also packaged the material and published it at Zenodo. The DOIs and references are listed in Table 3. The archive for the competition results includes the raw results in BenchExec's XML exchange format, the log output of the test generators and validator, and a mapping from files names to SHA-256 hashes. The hashes of the files are useful for validating the exact contents of a file, and accessing the files inside the archive that contains the test suites.

To provide transparent access to the exact versions of the test generators that were used in the competition, all tester archives are stored in a public Git repository. GitLab was used to host the repository for the tester archives due to its generous repository size limit of 10 GB. The final size of the Git repository is 1.47 GB.

Table 3: Artifacts published for Test-Comp 2020

| Content | DOI | Reference |
|---|---|---|
| Test-Generation Tasks | 10.5281/zenodo.3678250 | [7] |
| Competition Results | 10.5281/zenodo.3678264 | [6] |
| Test Suites (Witnesses) | 10.5281/zenodo.3678275 | [8] |

Table 4: Competition candidates with tool references and representing jury members

| **Participant** | **Ref.** | **Jury member** | **Affiliation** |
|---|---|---|---|
| CoVeriTest | [10, 31] | Marie-Christine Jakobs | TU Darmstadt, Germany |
| Esbmc | [22, 23] | Lucas Cordeiro | U. of Manchester, UK |
| HybridTiger | [15, 37] | Sebastian Ruland | TU Darmstadt, Germany |
| Klee | [17] | Martin Nowack | Imperial College London, UK |
| Legion | [36] | Gidon Ernst | LMU Munich, Germany |
| LibKluzzer | [34] | Hoang M. Le | U. of Bremen, Germany |
| PRTest | [35] | Thomas Lemberger | LMU Munich, Germany |
| Symbiotic | [18, 19] | Marek Chalupa | Masaryk U., Czechia |
| TracerX | [29, 30] | Joxan Jaffar | Nat. U. of Singapore, Singapore |
| VeriFuzz | [20] | Raveendra Kumar M. | Tata Consultancy Services, India |

## 5 Results and Discussion

For the second time, the competition experiments represent the state of the art in fully automatic test-generation for whole C programs. The report helps in understanding the improvements compared to last year, in terms of effectiveness (test coverage, as accumulated in the score) and efficiency (resource consumption in terms of CPU time). All results mentioned in this article were inspected and approved by the participants.

**Participating Test Generators.** Table 4 provides an overview of the participating test-generation systems and references to publications, as well as the team representatives of the jury of Test-Comp 2020. (The competition jury consists of the chair and one member of each participating team.) Table 5 lists the features and technologies that are used in the test-generation tools. An online table with information about all participating systems is provided on the competition web site.[16]

**Computing Resources.** The computing environment and the resource limits were mainly the same as for Test-Comp 2019 [5]: Each test run was limited to 8 processing units (cores), 15 GB of memory, and 15 min of CPU time. The test-suite validation was limited to 2 processing units, 7 GB of memory, and 5 h of CPU time (was 3 h for Test-Comp 2019). The machines for running the experiments are part of a compute cluster that consists of 168 machines; each test-generation run was executed on an otherwise completely unloaded, dedicated machine, in order

[16] https://sv-comp.sosy-lab.org/2020/systems.php

Table 5: Technologies and features that the competition candidates offer

| Participant | Bounded Model Checking | CEGAR | Evolutionary Algorithms | Explicit-Value Analysis | Floating-Point Arithmetics | Guidance by Coverage Measures | Predicate Abstraction | Random Execution | Symbolic Execution | Targeted Input Generation |
|---|---|---|---|---|---|---|---|---|---|---|
| CoVeriTest | | ✓ | | ✓ | ✓ | | ✓ | | | |
| Esbmc | ✓ | | | | ✓ | | | | | |
| HybridTiger | | ✓ | | ✓ | ✓ | | ✓ | | | |
| Klee | | | | | | | | | ✓ | ✓ |
| Legion | | | | | | ✓ | | ✓ | ✓ | ✓ |
| LibKluzzer | | | | | | ✓ | | ✓ | ✓ | |
| PRTest | | | | | | | | ✓ | | |
| Symbiotic | | | | | | ✓ | | | ✓ | ✓ |
| TracerX | ✓ | | | | | | | | ✓ | ✓ |
| VeriFuzz | ✓ | | ✓ | ✓ | | ✓ | | ✓ | | |

to achieve precise measurements. Each machine had one Intel Xeon E3-1230 v5 CPU, with 8 processing units each, a frequency of 3.4 GHz, 33 GB of RAM, and a GNU/Linux operating system (x86_64-linux, Ubuntu 18.04 with Linux kernel 4.15). We used BenchExec [12] to measure and control computing resources (CPU time, memory, CPU energy) and VerifierCloud [17] to distribute, install, run, and clean-up test-case generation runs, and to collect the results. The values for time and energy are accumulated over all cores of the CPU. To measure the CPU energy, we use CPU Energy Meter [13] (integrated in BenchExec [12]). Further technical parameters of the competition machines are available in the repository that also contains the benchmark definitions. [18]

One complete test-generation execution of the competition consisted of 29 899 single test-generation runs. The total CPU time was 178 days and the consumed energy 49.9 kWh for one complete competition run for test-generation (without validation). Test-suite validation consisted of 29 899 single test-suite

[17] https://vcloud.sosy-lab.org
[18] https://gitlab.com/sosy-lab/test-comp/bench-defs/tree/testcomp20

Table 6: Quantitative overview over all results; empty cells mark opt-outs

| Participant | Cover-Error 699 tasks | Cover-Branches 2531 tasks | Overall 3230 tasks |
|---|---|---|---|
| CoVeriTest | 405 | **1412** | 1836 |
| Esbmc | **506** | | |
| HybridTiger | 394 | 1351 | 1772 |
| Klee | 502 | 1342 | **2017** |
| Legion | 302 | 1257 | 1501 |
| LibKluzzer | **630** | **1597** | **2474** |
| PRTest | 66 | 545 | 500 |
| Symbiotic | 435 | 849 | 1548 |
| TracerX | 373 | 1244 | 1654 |
| VeriFuzz | **636** | **1577** | **2476** |

validation runs. The total consumed CPU time was 632 days. Each tool was executed several times, in order to make sure no installation issues occur during the execution. Including preruns, the infrastructure managed a total of 401 156 test-generation runs (consuming 1.8 years of CPU time) and 527 805 test-suite validation runs (consuming 6.5 years of CPU time). We did not measure the CPU energy during preruns.

**Quantitative Results.** Table 6 presents the quantitative overview of all tools and all categories. The head row mentions the category and the number of test tasks in that category. The tools are listed in alphabetical order; every table row lists the scores of one test generator. We indicate the top three candidates by formatting their scores in bold face and in larger font size. An empty table cell means that the tester opted-out from the respective main category (perhaps participating in subcategories only, restricting the evaluation to a specific topic). More information (including interactive tables, quantile plots for every category, and also the raw data in XML format) is available on the competition web site [19] and in the results artifact (see Table 3). Table 7 reports the top three testers for each category. The consumed run time (column 'CPU Time') is given in hours and the consumed energy (column 'Energy') is given in kWh.

**Score-Based Quantile Functions for Quality Assessment.** We use score-based quantile functions [12] because these visualizations make it easier to understand the results of the comparative evaluation. The web site [19] and the

[19] https://test-comp.sosy-lab.org/2020/results

Table 7: Overview of the top-three test generators for each category (measurement values for CPU time and energy rounded to two significant digits)

| Rank | Verifier | Score | CPU Time (in h) | Energy (in kWh) |
|---|---|---|---|---|
| ***Cover-Error*** | | | | |
| 1 | **VeriFuzz** | **636** | 17 | .22 |
| 2 | LibKluzzer | 630 | 130 | 1.3 |
| 3 | Esbmc | 506 | 9.5 | .11 |
| ***Cover-Branches*** | | | | |
| 1 | **LibKluzzer** | **1597** | 540 | 5.6 |
| 2 | VeriFuzz | 1577 | 590 | 7.5 |
| 3 | CoVeriTest | 1412 | 430 | 4.4 |
| ***Overall*** | | | | |
| 1 | **VeriFuzz** | **2476** | 610 | 7.7 |
| 2 | LibKluzzer | 2474 | 670 | 6.9 |
| 3 | Klee | 2017 | 460 | 5.2 |

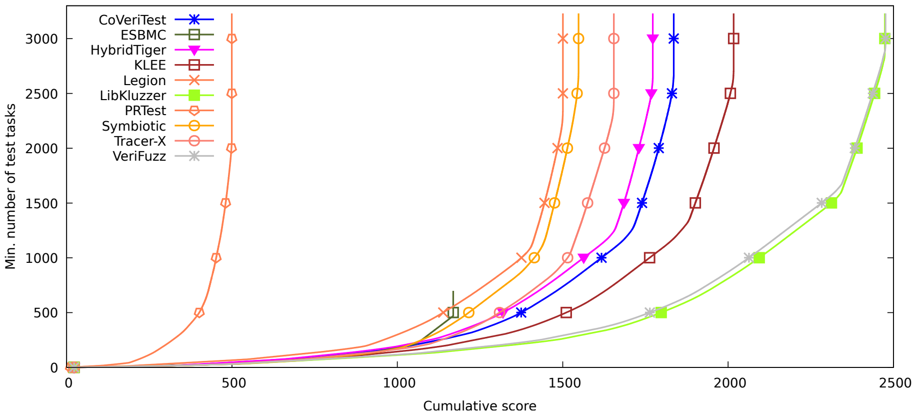

Fig. 4: Quantile functions for category *Overall*. Each quantile function illustrates the quantile ($x$-coordinate) of the scores obtained by test-generation runs below a certain number of test tasks ($y$-coordinate). More details were given previously [5]. A logarithmic scale is used for the time range from 1 s to 1000 s, and a linear scale is used for the time range between 0 s and 1 s.

Table 8: Alternative rankings; quality is given in score points (sp), CPU time in hours (h), energy in kilo-watt-hours (kWh), the rank measure in joule per score point (J/sp); measurement values are rounded to 2 significant digits

| Rank | Verifier | Quality (sp) | CPU Time (h) | CPU Energy (kWh) | Rank Measure (J/sp) |
|---|---|---|---|---|---|
| ***Green Testers*** | | | | | |
| 1 | Symbiotic | 1 548 | 41 | 0.50 | 1.2 |
| 2 | Legion | 1 501 | 160 | 1.8 | 4.4 |
| 3 | TracerX | 1 654 | 310 | 3.8 | 8.3 |
| worst | | | | | 53 |

results artifact (Table 3) include such a plot for each category; as example, we show the plot for category *Overall* (all test tasks) in Fig. 4. A total of 9 testers (all except Esbmc) participated in category *Overall*, for which the quantile plot shows the overall performance over all categories (scores for meta categories are normalized [2]). A more detailed discussion of score-based quantile plots for testing is provided in the previous competition report [5].

**Alternative Ranking: Green Test Generation — Low Energy Consumption.** Since a large part of the cost of test-generation is caused by the energy consumption, it might be important to also consider the energy efficiency in rankings, as complement to the official Test-Comp ranking. The energy is measured using CPU Energy Meter [13], which we use as part of BenchExec [12]. Table 8 is similar to Table 7, but contains the alternative ranking category *Green Testers*. Column 'Quality' gives the score in score points, column 'CPU Time' the CPU usage in hours, column 'CPU Energy' the CPU usage in kWh, column 'Rank Measure' uses the energy consumption per score point as rank measure: $\frac{\text{total CPU energy}}{\text{total score}}$, with the unit $J/sp$.

## 6 Conclusion

Test-Comp 2020, the 2nd edition of the Competition on Software Testing, attracted 10 participating teams. The competition offers an overview of the state of the art in automatic software testing for C programs. The competition does not only execute the test generators and collect results, but also validates the achieved coverage of the test suites, based on the latest version of the test-suite validator TestCov. The number of test tasks was increased to 3 230 (from 2 356 in Test-Comp 2019). As before, the jury and the organizer made sure that the competition follows the high quality standards of the FASE conference, in particular with respect to the important principles of fairness, community support, and transparency.
